# Performance Evaluation and Compensation Method of Trigger Probes in Measurement Based on the Abbé Principle

**DOI:** 10.3390/s20082413

**Published:** 2020-04-23

**Authors:** Guoying Ren, Xinghua Qu, Xiangjun Chen

**Affiliations:** 1 State Key Laboratory of Precision Measurement Technology and Instruments, Tianjin University, Tianjin 300072, China; quxinghua@tju.edu.cn (X.Q.); jun689074@163.com (X.C.); 2 Length Division, National Institute of Metrology, Beijing 100029, China

**Keywords:** manufacturing metrology, quality control, trigger-probe, CMM, machine tool metrology, calibration

## Abstract

Trigger probes are widely used in precision manufacturing industries such as coordinate measuring machines (CMM) and high-end computer numerical control(CNC) machine tools for quality control. Their performance and accuracy often determine the measurement results and the quality of the product manufacturing. However, because there is no accurate measurement of the trigger force in different directions of the probe, and no special measuring device to calibrate the characteristic parameters of the probe in traditional measurement methods, it is impossible to exactly compensate for the measurement error caused by the trigger force of the probe in the measurement process. The accuracy of the measurement of the equipment can be improved by abiding by the Abbé principle. Thus, in order to better evaluate the performance parameters of the probe and realize the accurate compensation for its errors, this paper presents a method which can directly measure the performance parameters of the trigger probe based on the Abbé measurement principle, expounds the measurement principle, the establishment of the mathematical model, and the calibration system, and finishes with an experimental verification and measurement uncertainty analysis. The experimental results show that this method can obtain the exact calibration errors of the performance parameters of the trigger probe intuitively, realize the compensation for the errors of the probe in the measurement process, and effectively improve the measurement accuracy.

## 1. Introduction

Trigger probes are widely used in coordinate measuring machines (CMM), high-end computer numerical control(CNC) machine tools, and other precision manufacturing applications, and are an important component of coordinate measuring machines and CNC machine tools. The performance of a trigger probe directly affects the measurement accuracy of the CMMs and the quality of the products manufactured by CNC machines.

With the higher requirements for manufacturing quality and the technological changes brought about by Industry 4.0, the manufacturing of trigger probes tends to be standardized and modularized, and the probes can be used independently like a sensor. This makes it possible to calibrate the probe separately. The performance parameters of trigger probes include repeatability of trigger position; maximum value, minimum value, and change of pre-travel; trigger hysteresis (drift of zero position); anisotropic deviation. Through the calibration of the performance parameters of the probe, the measurement error of the probe can be compensated for to reduce the probing error and improve the measurement accuracy [1,2], which is particularly significant when using a long measuring rod.

In order to calculate and compensate for the influence of probe error, Woźniak and Dobosz [3,4,5,6,7] proposed a three-dimensional theoretical model of a touch triggered probe, described and simulated its measurement feasibility, and performed experimental verification. However, this method inevitably brings errors to the measurement results due to the use of a contact force displacement transducer (although it is emphasized that the measuring force is very small) to determine the contact position and trigger position during the contact of the probe and the tested part.

Based on the analysis of the working mechanism of the probe, Zhao et al. [8,9] established a mathematical model and calibration device for the pre-travel of the probe, and measured and analyzed the pre-travel characteristics of the probe. However, this method has limitations, because it requires that the probe ball tip must be a conductive metal. In fact, a large number of probe balls used in precision manufacturing are ruby materials, which are not conductive. 

Li et al. [10] proposed a dynamic model to describe the probe measurement process in the time domain. However, the pre-travel in this method is based on theoretical calculation rather than actual measurement.

In addition, similar experimental studies of probe error and compensation can be found. Shen et al. describe a pre-travel model and a trigger force model for touch trigger probes to obtain the value of pre-travel and experimental validation [11]; P. A. Cauchick Miguel et al. [12,13] report the error sources of probe performance [14,15] and the development of a test apparatus to be used for checking touch trigger probes, which uses a gauge block surface to contact the probe ball tip until the probe is triggered, at which point the coordinate position is displayed by laser.Mayer et al. [16] proposes a 3D error correction model and an experimental method based on the use of a micrometer and a high magnification video camera. Li et al. [17] proposes a compensation method for probe errors using a 3D error map based on the probe’s mechanical model. Park et al. [18] introduces a touch probe to estimate the pre-travel, and Ma et al. [19] describes a pre-travel error model of a trigger probe and its simulation. Cai et al. [20] builds a mathematical model of pre-travel error to reveal its anisotropy.

The above methods are all based on the change of electronic voltage or force on the probe to measure the pre-travel, rather than directly monitoring the displacement change of the probe ball tip to calibrate the probe parameters. Thus, this approach calculates the difference between the position of the trigger signal and the position of the actual measurement point based on the direct measurement of the trigger force in the measurement process. In addition, the above measurement methods do not conform to the Abbé measurement principle. As a result, there is a research gap for further improvement.

Based on the analysis of the above methods, this paper proposes a new method based on the Abbé measurement principle [21] to directly measure the parameter error of the trigger probe, which can directly obtain the calibration error of each performance parameter of the probe, and realize the error compensation of the measurement value in the measurement process.

## 2. Methodology and Measurement Method

### 2.1. Abbe’s Principle

Abbé’s principle relates to accuracy when measuring dimensions. The principle is also an important guideline for designing measuring instruments. This principle states that, “In order to improve measurement accuracy, the measurement target and the scale of the measuring instrument must be placed in a collinear fashion in the measurement direction”.

**Figure 1:**
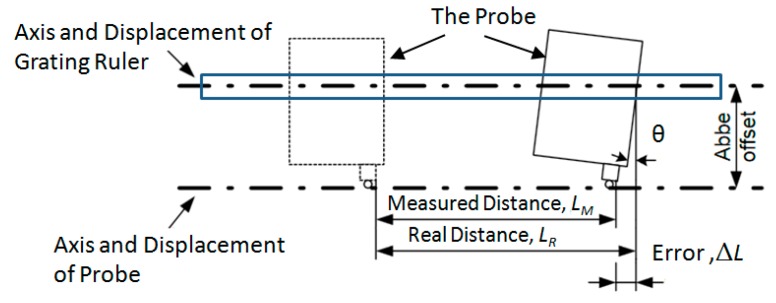
An illustration of an Abbé error.

An Abbé error occurs when, during measurements, the measured probe does not move perfectly straight along the guide line and there appear to be angular movements, which cause sloping of the measured probe. The sloping of the measured probe is greater the longer the distance between the movement axis of the grating ruler and the axis of probe movement. This distance is called an Abbé offset(see Figure 1).

The distance error Δ*L* is calculated by the measured distance *L_M_* of the probe and the real distance *L_R_* of the grating ruler calibrated by a laser interferometer, according to:

(1) $$\Delta L=L_{R}-L_{M}$$

An Abbé error may be avoided only when there are no angular θ movements and no Abbé offset of the grating ruler and the axis of probe.

### 2.2. Measurement Method

**Figure 2:**
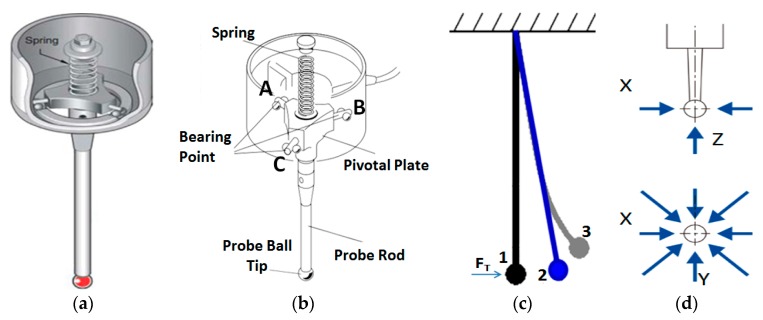
Structure of the touch trigger probe: (**a**) 3D structure drawing of the probe; (**b**) schematic diagram of each part of the probe; (**c**) stress deformation diagram of the probe under the trigger force *F_T_*; (**d**) diagram of five degrees of freedom distribution of the probe.

From the structure of the trigger probe [19], we can see that in order to achieve the calibration of the probe’s pre-travel and trigger errors, it is necessary to directly measure the displacement change of the probe ball tip, so as to effectively avoid the elastic deformation error and Abbé error caused by the probe rod, as shown in Figure 2. 

As can be seen from Figure 2, Figure 2a shows a 3D model of the trigger probe. Figure 2b shows the internal mounting structure of the trigger probe, and three supporting points. A,B and Care evenly distributed in a circular plane, with an angle of 120° between any two points. In Figure 2c, 1indicates the position of the probe in the free state without the trigger force *F_T_*, 2 represents the ideal non-deformation position of the probe under the trigger force *F_T_*, and 3 represents the actual position of the probe after stress deformation. Figure 2d shows that the probe has three directions, namely, X,Y,Z and five degrees of freedom in the directions of +X, −X, +Y, −Y, +Z.

**Figure 3:**
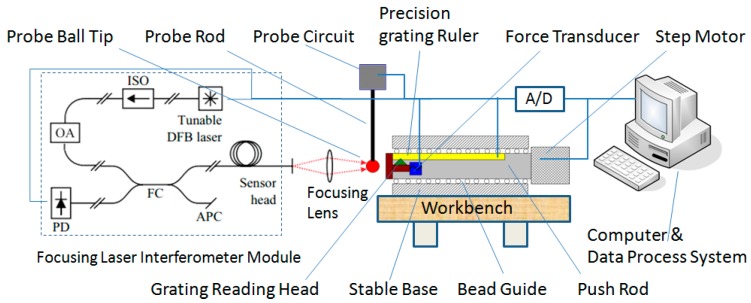
A schematic diagram of the probe parameter calibration system.

In order to effectively measure the displacement change of the probe ball tip, the measurement principle established in this paper is shown in Figure 3. The movement axis of the grating ruler and the axis of probe movement are collinear in the measurement direction. The probe is fixed on a workbench. The computer controls the step motor to drive the push rod to move towards the probe at a certain speed, and the displacement is measured by the precise grating ruler. The displacement of the probe ball tip is measured by a laser focusing displacement sensor based on the principle of laser interference displacement measurement [22,23], which focuses the measurement light emitted by the laser on the probe ball tip, and the probe ball tip then reflects the measurement light to the laser head. After the signal processing inside the laser head, the displacement change of the probe ball tip can be obtained.

**Figure 4:**
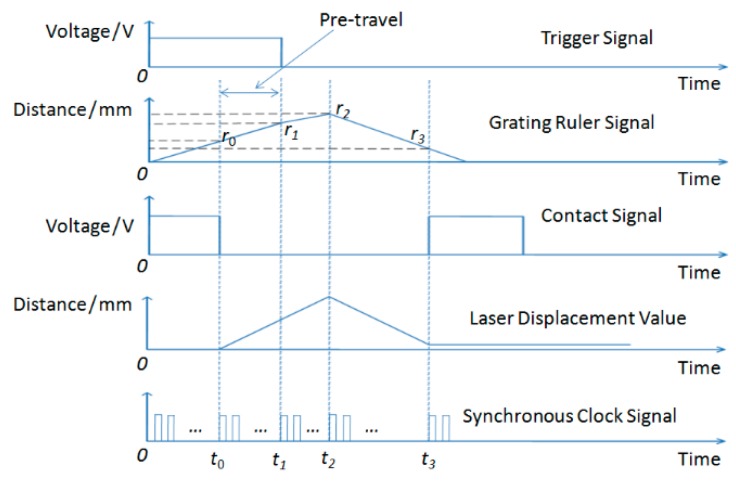
Measurement time sequence diagram of the calibration system.

The measurement process is as follows, and the time sequence change of the calibration system during measurement is shown in Figure 4. Before calibration, the push rod does not touch the probe ball tip. At the beginning of calibration, the computer controls the step motor to drive the push rod forward to approach the probe ball tip, assuming that the value of the grating ruler fixed on the push rod at the moment the motor starts is zero. Because the laser is used to monitor the change of probe position, the displacement value of the laser interferometer does not change before the push rod touches the probe ball tip, assuming that the laser value is zero at this time. The pushrod continues to be driven forward until it just contacts the probe ball tip, the laser value begins to become non-zero, and the contact signal changes from high level to low level. The time of the contact instant is recorded as *t*_0_ and the grating displacement value as *r*_0_; meanwhile, the time and the value are sampled and stored in the computer. The push rod continues to move forward until the trigger signal of the probe occurs; the trigger signal then changes from high level to low level, and the computer records the time of the trigger moment *t*_1_ and the grating ruler displacement *r*_1_. When the computer control system receives the trigger signal from the probe circuit, it stops the step motor immediately according to the control program. Because of the control strategy and the existence of machine inertia, the push rod will still push the probe ball tip a certain distance (the smaller the inertia, the smaller the distance) forward; the system records and stores the grating ruler displacement as *r*_2_ and the time at the moment the push rod stops as *t*_2_. Then, the computer controls the motor to drive the push rod to return to its zero position. Because of the mechanical characteristics of the trigger probe, the probe cannot return to its initial zero position, so there will be a return-to-zero error. When the laser displacement value does not change, the grating displacement *r*_3_ and the time *t*_3_ are recorded and stored, and the contact signal changes from low level to high level.

Since the probe has three directions and five degrees of freedom, the parameters of the trigger probe in any direction can be calculated as follows:

The pre-travel of the probe is:

(2) $$L_{\mathrm{Pr}e}=r_{1}-r_{0}$$

Trigger hysteresis (drift of zero position):

(3) $$\Delta S_{Hyst}=r_{3}-r_{0}$$

Trigger position repeatability (unidirectional repeatability)when the number of measurements is *n*:

(4) $$\Delta R_{TrigPos}=\max(r_{11},r_{12}\ldots r_{1i})-\min(r_{11},r_{12}\ldots r_{1i}),i\in [1,n]$$

According to the above definition, we can easily calculate the anisotropic deviation of pre-travel, trigger hysteresis, and trigger position repeatability.

It can be seen from Figure 4 that the value*r*_1_ is the displacement of grating ruler under the compression condition of the tested part and the probe ball tip at the trigger moment of the probe, which is highly important and complex (see Section 3 for details). In the following section, the calculation process of *r*_1_ will be emphatically described by taking the measurement of the pre-travel as an example.

## 3. Mathematical Modeling and Simulation

**Figure 5:**
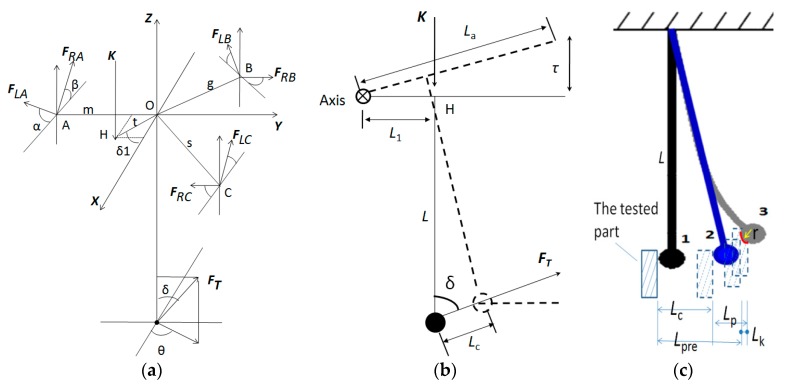
Displacement change and stress analysis diagram of the probe in the process of triggering. (**a**) force analysis in the process of probe triggering; (**b**) equivalent diagram of force and rigid displacement change of the probe in the process of probe triggering; (**c**) displacement change of each stage in the process of probe triggering.

According to Figure 2b,c, the coordinate system is established as shown in Figure 5, and the force and displacement changes of the probe are analyzed in the process from contacting the measured part to sending out the trigger signal [24].

In Figure 5a, ***F*_Li_** (i = A, B, C) is the support force of the right support ball to the probe positioning pin at point I; ***F*_Ri_** is the support force of the left support ball to the probe positioning pin at point i; α is the included angle of ***F*_LA_** and the connection line of the two supporting ball centers at point A; β is the included angle of ***F*_RA_** and the connection line of the two supporting ball centers at point A. The included angles at point B and point Care the same as that at point A. ***K*** is the sum of the spring pre-pressure force on the probe and the probe gravity. t is the distance between the force ***K*** and the center of the mass of the probe, that is, the eccentricity. δ_1_ is the angle between the line $\bar{OH}$ from the gravity point O of the probe to the point H of the force ***K*** and the positive direction of the X axis. The distance between point A and the center of the probe is m; the distance between point B and the center of the probe is g; the distance between point C and the center of the probe is s; the distance between the center of the probe and the center of the probe ball is *L*.***F*_T_** is the measuring force when the probe contacts the tested part to generate the trigger signal; δ is the angle between the force ***F*_T_** and the positive direction of the Z axis; θ is the angle between the projection line of the force ***F*_T_** on the XY plane and the positive direction of the X axis; r is the radius of probe ball tip.

In Figure 5b,*τ*is the rotation distance of a point of the point A, point B and point C around the rotation axis comprised of two other points under the action of force ***F*_T_**, and *L_a_* is the equivalent length from the point of *τ* to the rotation axis.

In Figure 5c, *L*_c_ is the rigid displacement from the idle state of the probe to the trigger moment; *L_p_* is the deflection displacement of the probe from the idle state of the probe to the trigger moment; *L_k_* is the elastic compression displacement of the contact between the probe and the tested part when the force is ***F*_T_**.

According to Figure 5c, the pre-travel $L_{pre}$ of the probe can be calculated according toEquation (5):

(5) $$L_{pre}=L_{c}+L_{p}-L_{k}$$

### 3.1. Rigid Displacement, L_C_

Considering the probe as a rigid body, when the probe reaches the force balance state at the trigger moment, according to the static balance concept, the combined external force and the combined external moment are zero, as shown in Figure 5a [25].

When the probe is triggered, the force of a certain point of the probe will change from $F_{Li}$ or $F_{Ri}$ (i = A, B, C) to zero in Figure 5b. According to the elastic mechanics theory, the distance *τ* can be calculated according to Equation (6):

(6) $$\tau =0.655\times n_{\delta }\times \sqrt[{3}]{{\left(\frac{1-\mu_{1}^{2}}{E_{1}}+\frac{1-\mu_{2}^{2}}{E_{2}}\right)}^{2}\cdot \frac{2R_{2}+R_{1}}{R_{2}R_{1}}\times F_{T}{}^{2}}$$

where $n_{\delta }$ is the contact coefficient; $\mu_{1}$ and $\mu_{2}$ are the Poisson’s ratio of the support ball and the positioning pin, respectively; *E*_1_ and *E*_2_ are the modulus of elasticity of the support ball and the positioning pin, respectively;*R*_1_ and *R*_2_ are the radius of the support ball and the positioning pin, respectively.

When the displacement of a certain point of point A, point B and point C is the elastic deformation *τ*, the displacement change of the probe ball tip can be calculated according to Equation (7):

(7) $$L_{c}=\frac{\tau }{L_{a}}\times L=\frac{L}{L_{a}}\times 0.655\times n_{\delta }\times \sqrt[{3}]{{\left(\frac{1-\mu_{1}^{2}}{E_{1}}+\frac{1-\mu_{2}^{2}}{E_{2}}\right)}^{2}\cdot \frac{2R_{2}+R_{1}}{R_{2}R_{1}}\times F_{T}{}^{2}}$$

### 3.2. Deflection Displacement, Lp

According to material mechanics, the deflection of the cantilever beam is as follows:

(8) $$L_{p}=\frac{F_{T}x^{2}}{6EI}\left(3L-x\right)$$

where *L* is the length of the probe rod; *E* is the modulus of elasticity of the probe rod; *x* is the lengthfrom the root point H (see Figure 5b) of the probe rod to the calculated point toward the direction of the probe ball tip (e.g., *x* equals *L* when the calculated point is the center of the probe ball tip); *L_p_* is the deflection of the probe rod; *I* is the moment of inertia of the probe rod cross section face to the center axis. For a circular probe rod, the value of *I* can be calculated according to Equation (9):

(9) $$I=\frac{\pi d^{4}}{64}$$

where d is the diameter of the probe rod.

Then, the deflection value at the position of the probe ball tip can be calculated according toEquation (10):

(10) $$L_{p}=\frac{F_{T}L^{3}}{3EI}=\frac{64L^{3}}{3\pi Ed^{4}}\times F_{T}$$

### 3.3. Elastic Compression Displacement, L_k_

Since the contact between the probe and the tested part belongs to the contact type of the ball tip and the plane, the contact deformation *L_k_*can be calculated according to Equation (11):

(11) $$L_{k}=0.8255\times \sqrt[{3}]{{\left(\frac{1-\mu_{3}^{2}}{E_{3}}+\frac{1-\mu_{4}^{2}}{E_{4}}\right)}^{2}\cdot \frac{F_{T}{}^{2}}{r}}$$

where $\mu_{3}$ and $\mu_{4}$ are the Poisson’s ratio of the probe ball tip and the tested part, respectively; *E*_3_ and *E*_4_ are the modulus of elasticity of the probe ball tip and the tested part, respectively; *r* is the diameter of the probe ball tip.

### 3.4. The Pre-Travel of the Probe, Lpre

According to Equation (2), under the action of force *F*_T_, the displacement change at the position of the probe is computed according to Equation (12):

(12) $$\begin{array}{c} L_{pre}=L_{c}+L_{p}-L_{k}=\frac{L}{L_{a}}\times 0.655\times n_{\delta }\times \sqrt[{3}]{{\left(\frac{1-\mu_{1}^{2}}{E_{1}}+\frac{1-\mu_{2}^{2}}{E_{2}}\right)}^{2}\cdot \frac{2R_{2}+R_{1}}{R_{2}R_{1}}\times F_{T}{}^{2}}\\ +\frac{64L^{3}}{3\pi Ed^{4}}\times F_{T}-0.8255\times \sqrt[{3}]{{\left(\frac{1-\mu_{3}^{2}}{E_{3}}+\frac{1-\mu_{4}^{2}}{E_{4}}\right)}^{2}\cdot \frac{F_{T}{}^{2}}{r}} \end{array}$$

It can be seen from Equation (12) that the pre-travel is inversely proportional to the elastic modulus *E* of the material of the probe and the tested part, directly proportional to the trigger force *F_T_* and the length *L* of the probe rod, and less affected by the Poisson’s ratio *μ*of the material. It is also related to the contact radius*R*_1_, *R*_2_, and *r* of the material.

### 3.5. Modelling Simulation

**Figure 6:**
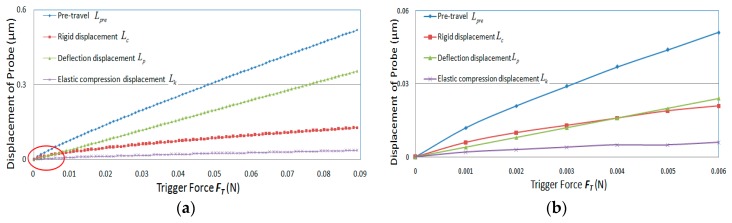
Simulation diagram of pre-travel and its components changing with trigger force. (**a**) various displacement changes in the whole trigger force range; (**b**) enlarged view of the red marked area in Figure 6a.

According to the above mathematical model, and considering that the trigger force given by the manufacturer is generally less than 0.2 N, the mathematical model was simulated based on Matlab software [26], and the type and parameters of the probe and the selected materials were the same as those used in the Section 4. The simulation results are shown in Figure 6.

From the simulation results(see Figure 6), it can be seen that when the measuring force is less than 0.003 N, the pre-travel, *L_pre_*, of the probe mainly comes from the rigid displacement caused by the measuring force; when the measuring force is greater than 0.003 N, the bending deflection, *L_p_,* of the probe rod of the probe begins to become the main reason for the increase in the pre-travel of the probe, while the elastic compression deformation, *L_k_*, of the probe during the whole triggering process is basically unchanged. Therefore, when designing the probe, material with a large modulus of elasticity,*E*_4_,should be preferred to make the probe rod and, in addition, the trigger force *F_T* should be minimized as much as possible.

## 4. Device and Experiments

**Figure 7:**
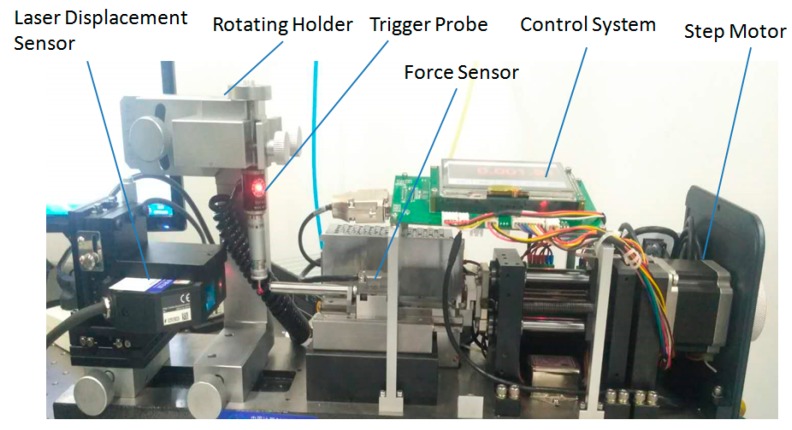
A picture of the probe parameter calibration system.

According to the description of measurement principle in Figure 3, the calibration system of the parameters of the trigger probe was established, as shown in Figure 7. Taking a type of trigger probe widely used in the industry as the test object, the pre-travel and unidirectional repeatability of the probe were measured to verify the function of this calibration system.

For the probe, the relevant parameters are as follows:

$n_{\delta }$ = 0.9744, $\mu_{1}$ = $\mu_{2}$ = 0, $\mu_{3}$ = $\mu_{4}$ = 0, $E_{1}$ = $E_{2}$ = E = 200 kN/mm^2^, $R_{1}$ = $R_{2}$ = 1.000 mm, L = 18 mm, L/$L_{a}$ = 1.5, d = 2 mm, $E_{3}$ = 620 kN/mm^2^, $E_{4}$ = 200 kN/mm^2^, r = 4 mm.

According to Equation (11), the pre-travel of the probe is computed according to Equation (13).

(13) $$\begin{array}{c} L_{pre}=L_{c}+L_{p}-L_{k}=0.0000641\times F_{T}{}^{2/3}+0.012376F_{T}-0.0000183\times F_{T}{}^{2/3}\\ =0.0000458\times F_{T}{}^{2/3}+0.012376F_{T} \end{array}$$

Suppose, $f(F_{T})=L_{pre}=0.0000458\times F_{T}{}^{2/3}+0.012376F_{T}$, then,

(14) $$F_{T}=f^{\prime }(L_{pre})$$

When the probe is triggered, the elastic compression displacement *L_k_* is calculated according to Equations (11) and (15):

(15) $$L_{k}=0.0000183\times F_{T}{}^{2/3}=0.0000183\times f^{\prime }{\left(L_{pre}\right)}^{2/3}$$

**Figure 8:**
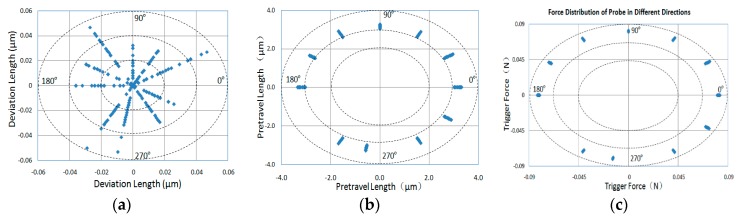
Error distribution of probe parameter calibration in different directions. (**a**) the repeatability error distribution of the trigger position of the probe; (**b**) the pre-travel distribution of the probe; (**c**) the measurement force distribution of the probe at the moment of trigger.

According to the above analysis, the measurement results of the probe based on the above method can be compensated for in the measurement data when the pre-travel behavior occurs, because the moment is the time when the probe just contacts the test part, and the compensated data should be closer to the real value of the test part. The above analysis can be applied to any angle direction of the probe. Taking the evaluation of the probe’s pre-travel characteristics in the XY plane as an example, and measuring the probe at different angle intervals within 360° in the XY plane, the measurement results of the parameters of the probe are shown in Figure 8. 

From Figure 8a, we can see that the error range of the trigger repeatability of the probe is different in different directions. The error range in the direction of angles60°and 90° is very small (the smallest error range is 0.03 μm), and the error range in the directions of 30°,120°, 240°and 260°is very big (the largest error range is 0.058 μm). The average error of the trigger repeatability is 0.02 μm.

From Figure 8b, we can see that the variation of pre-travel of the probe is also different in different directions. The smallest variation is 0.24 μm, which is in the direction of angle 90°, and the largest is 0.41 μm in the direction of angle 30°. The average of the pre-travel is 3.17 μm.

From Figure 8c, we can see that the variation of trigger force of the probe is also different in different directions. The smallest variation is 0.002 N which is in the direction of angle 90°, and the largest is 0.005 N in the direction of angle 30°. The average of the trigger force is 0.082 N.

According to the principle of the calibration system (see Figure 3), because the speed of signal acquisition and the latch are at the nanosecond level, the displacement measurement accuracy of the system mainly depends on the accuracy of the grating ruler. 

**Figure 9:**
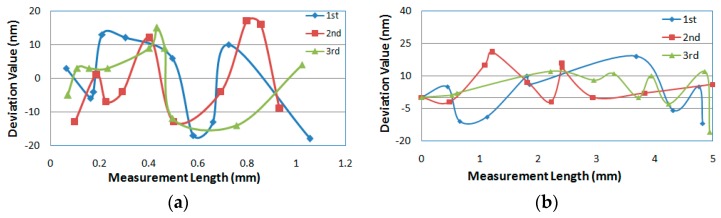
Error distribution of length measurement of the probe parameter calibration system. (**a**) error distribution within 1 mm; (**b**) error distribution within 5 mm.

In order to verify the displacement measurement accuracy of the calibration system, a laser interferometer is installed on the measuring line of the calibration system, which ensures that the measurement process conforms to the Abbé principle to minimize the measurement error sources. The test results in the range of 0–1 mm are shown in Figure 9a. When the probe is not installed in the exact range of 0–1 mm, but at any other position within 5 mm of the guide rail travel of the calibration system, the test results are shown in Figure 9b.

From Figure 9a, we can see that the variation trend of the deviation value of the three measurement results is basically the same. However, in Figure 9b, we see that the variation trend of the deviation value of the three times measurement results is not the same, which may be due to a slight random drift of the accuracy correction of the calibration device over time over a large length. However, it is clear from Figure 9 that the maximum error of the displacement measurement of the device is less than 20 nm.

In order to verify the correctness of the above mathematical model and the overall accuracy of the device, the probe with its performance parameters calibrated by the above method was installed on a CMM to measure [2] the diameter of the standard sphere with a known truth value of 29.98604 mm (this value was obtained by the Labconcept Nano 350 instrument manufactured by the Trimos company in Switzerland, and the measurement uncertainty of this instrument is 0.07+L/2000μm, L:mm) along a certain direction. The trigger force of this probe was the above mentioned 0.082 N, and the measurement error caused by the trigger force was compensated for in the diameter measurement value. The measurement results are shown in Table 1.

From Table 1, it can be seen that the error between the truth value of the standard sphere diameter and the actual measured value of the diameter before compensation is −0.84 μm, and the difference between the truth value of the standard sphere diameter and the compensated value of the diameter is −0.04 μm. This shows that the algorithm proposed in this paper is correct and valid for the measurement, and can improve the measurement accuracy.

## 5. Measurement Uncertainty

From this device, we can evaluate the uncertainty in measurement in accordance with the GUM(“Guide to the Expression of Uncertainty in Measurement, ”ISO, Switzerland, 1993, corrected and reprinted 1995). The calibration value is obtained by Equation (16) [27,28]:

(16) $$L=L_{0}-\alpha \cdot \Delta T\cdot L_{0}+\varepsilon_{0}+\varepsilon_{Tr}$$

where:

*L*:the measured distance of the probe at the reference temperature of 20 °C.

*L*_0_:the displacement measured by the grating ruler.

α:the thermal expansion coefficient of the grating ruler.

Δ*T*: the difference between the grating ruler temperature and the reference temperature of 20 °C.

$\varepsilon_{0}$:repeatability deviation at the zero position in measurement.

$\varepsilon_{Tr}$:repeatability deviation at the trigger position in measurement.

The uncertainty of the probe calibration is calculated by Equation (17):

(17) $$u{\left(L\right)}^{2}=u{\left(L_{0}\right)}^{2}+u{\left(\alpha \right)}^{2}\cdot \Delta T\cdot L_{0}+u{\left(\Delta T\right)}^{2}\cdot \alpha \cdot L_{0}+u{\left(\varepsilon_{0}\right)}^{2}+u{\left(\varepsilon_{Tr}\right)}^{2}$$

Table 2 shows the uncertainty components in the probe calibration.

In Table 2,1–6 are the independent sources of uncertainty, and 7–10 are dependent on thermal expansion uncertainties and have been converted to the length *L*.

It can be seen from Table 2 that the repeatability errors of the trigger position and the zero position are the largest error sources independent of the measurement length, and are mainly related to the probe’s mechanical mechanism, particularly the probe’s rigidity and mechanical reset function. Among the error sources related to the measurement length, temperature is the largest, which means that the temperature should be controlled as much as possible to ensure small deviations in uniformity and fluctuations.

Then, the uncertainty in measurement of the trigger probe calibration using this device is as follows:

(18) $$U=(k\times \sqrt{0.0999^{2}+{\left(0.129L\right)}^{2}})\approx (k\times \sqrt{0.10^{2}+{\left(0.13L\right)}^{2}})µm;$$

where:

*L*: the measurement length, unit m; 

*k*:coverage factor [27]; this is generally equal to 2, representing 95% confidence probability for the measurement result.

## 6. Conclusions

In order to accurately measure and calibrate the parameters of a trigger probe, this paper proposes a calibration method based on the Abbé principle and develops a calibration device that can directly measure the displacement change of the probe ball tip and simultaneously collect all signals based on a high-speed data acquisition circuit. After data processing, it is convenient to calculate the parameter errors of the trigger probe, such as the pre-travel and other parameter errors.

It can be seen from the measurement results and uncertainty analysis that this method can effectively meet the calibration requirements of trigger probes, and provide technical support for the precision calibration of trigger probe in CMMs and CNC machine tools in industrial applications.

If a user or manufacturer wants to improve the measurement accuracy of a CMM with the trigger probe, the probe error in different directions of the probe can be first measured according to this method before use, and the measured error parameters can then be written into the CMM as the system parameters of the probe to facilitate the real-time error compensation of the measurement results. If it is not convenient to write the parameters into the CMM, they can also be manually computed by the user to compensate the measuring results after measurement.

Although the mathematical model of the calibration system effectively estimates various parameters of the trigger probe and their influence, the application of the model and calibration method is limited to the trigger probe of this kind of mechanical structure.

In the future, our work will focus on reducing the measurement uncertainty and improving the calibration accuracy according to the error sources. It is also hoped that this paper will provide a new measurement method reference for related researchers.

## Figures and Tables

**Table 1:** The experimental data of the diameter of the standard sphere measured by the coordinate measuring machine (CMM) with the probe.

No.	Actual Measured Diameter Value (mm)	Compensated Diameter Value (mm)	Truth Value of the Diameter (mm)
1	29.9853	29.9860	29.98604
2	29.9845	29.9857	
3	29.9857	29.9864	
4	29.9849	29.9856	
5	29.9852	29.9859	
6	29.9854	29.9861	
7	29.9856	29.9863	
8	29.9851	29.9858	
9	29.9843	29.9860	
10	29.9855	29.9858	
Average value	29.9852	29.9860	
Error to nominal diameter	−0.00084	−0.00004	

**Table 2:** budget in probe calibration.

No.	Sources of Uncertainty	Magnitude	Type	Uncertainty
1	Probing	0.034 μm	A	0.034 μm
2	Calibration of probe	0.030 μm	B	0.030 μm
3	Uncertainty of standard sphere	0.030 μm	B	0.030 μm
4	Accuracy of grating scale displacement measurement	0.020 μm	B	0.020 μm
5	Repeatability deviation at zero position	0.055 μm	A	0.055 μm
6	Repeatability deviation at trigger position	0.060 μm	A	0.060 μm
7	Temperature measurement	5 mK	B	0.058 Lμm
8	Temperature distribution	10 mK	A	0.115 Lμm
9	Thermal expansion coefficient	1.0 × 10^−6^/K	B	0.010 Lμm
10	Error of cosine (gauge)	0.1 mm/100 mm	B	0.001 Lμm
